# Exploring molecular pathology of chronic kidney disease in systemic sclerosis by analysis of urinary and serum proteins

**DOI:** 10.1093/rap/rkaa083

**Published:** 2021-01-07

**Authors:** Edward P Stern, Robert Unwin, Aine Burns, Voon H Ong, Christopher P Denton

**Affiliations:** 1 UCL Centre for Rheumatology and Connective Tissue Diseases; 2 UCL Department of Renal Medicine, UCL, London; 3 AstraZeneca BioPharmaceuticals R&D, Early CVRM, Cambridge, UK

**Keywords:** renal, biomarker, scleroderma, systemic sclerosis, chemokine, adhesion molecule

## Abstract

**Objective.:**

Renal involvement is common in systemic sclerosis (scleroderma; SSc) and includes chronic kidney disease (CKD). We have performed analysis of urinary proteins to gain insight into local molecular pathology of CKD in SSc and identify candidate markers for use in clinical trials.

**Methods.:**

To evaluate urinary proteins that might specifically reflect SSc-related CKD, patients were recruited with confirmed SSc and stratified for the presence or absence of CKD. Controls included patients with CKD and no SSc, in addition to healthy volunteers. Candidate markers were measured in serum and urine by multiplex immunoassay testing for IL6, IL18, TNF-α, monocyte chemoattractant protein 1 (MCP1), monocyte chemoattractant protein 3 (MCP3), VEGF and the soluble adhesion molecules vascular cell adhesion molecule 1 (VCAM-1) and intercellular adhesion molecule 1 (ICAM-1).

**Results.:**

One hundred and two subjects were examined, including patients with SSc with no evidence of CKD (*n* = 40), SSc with CKD (*n* = 39), non-SSc CKD (*n* = 11) and healthy volunteers (*n* = 12). Urinary levels of IL6, MCP1, TNF-α, MCP3, IL18 and ICAM-1 were elevated in SSc patients compared with healthy controls. The most significant differences were for MCP1 and ICAM-1 (both *P* < 0.0001), and these analytes also showed the most significant differences between groups overall (*P* = 0.003 for MCP1 and *P* < 0.0001 for ICAM-1). These markers showed a trend (MCP1, *P* = 0.0868) or a significant difference (ICAM-1, *P* = 0.0134) between SSc–CKD and SSc with normal renal function.

**Conclusion.:**

Urinary levels of candidate molecular markers appear to reflect SSc–CKD more than serum markers. MCP1 and ICAM-1 are promising molecular markers for SSc–CKD and might be potential biomarkers of SSc renal involvement. This might be explored in future prospective analyses.

**Key messages**

- Elevated inflammatory proteins in serum of SSc patients reflect disease biology at multiple sites.
- In SSc, urinary proteins can provide insight into local disease mechanisms in chronic kidney disease.
- This study identifies two candidate renal biomarkers, urinary ICAM-1 and MCP1, that might be specific for SSc.

## Introduction

Systemic sclerosis (scleroderma; SSc) is a multi-system disease with high mortality owing to the involvement of vital organs, including the heart, lungs, gut and the renal tract [1]. Renal complications can be serious, and in the past, accelerated phase hypertension and acute kidney injury attributable to scleroderma renal crisis (SRC) was the most frequent cause of death in SSc [2]. The overall outcome of SRC has improved owing to routine use of angiotensin-converting enzyme inhibitors in managing the acute episode of SRC and better supportive care for the longer-term consequences [3]. The pathobiology of SRC is incompletely understood, although recent genetic and histological analyses identify potential molecular mechanisms relevant to susceptibility and pathogenesis [4].

Although recovery from the acute kidney injury of SRC is often excellent, SRC remains an important mechanism leading to chronic kidney disease (CKD) in SSc. In addition, CKD occurs in a large proportion of SSc patients because of systemic vasculopathy, fibrosis and other mechanisms, such as overlap CTD, including SLE and vasculitis. Other pathologies, such as interstitial nephritis or drug toxicity, can also contribute to CKD in SSc. Previous studies of sequential unselected patients suggested that CKD is present in ≤50% of SSc cases [5]. Although often presenting as mild renal impairment that is not of immediate clinical importance, CKD has implications for long-term renal and patient outcomes and is a major determinant of the long-term outcome after SRC.

Creatinine-derived measures of glomerular filtration rate (GFR) deteriorate only once there is significant parenchymal abnormality in the kidney; therefore, there is a general need for more sensitive markers of disease activity in CKD, including the context of SSc. Such tests might also help to discriminate SSc from non-SSc-related processes in the kidney in order that management could be more appropriate. In addition, new markers could help in distinguishing clinically important or progressive CKD from more stable cases. Easily measured biomarkers could be applied to outcome assessment in clinical trials or practice and might provide early indicators of therapeutic response that could predict future clinical benefit.

There have been many recent studies exploring potential biomarkers in SSc, and these have focused on examination of skin biopsies or peripheral blood, including cell-based approaches such as gene expression, in addition to methods examining serum, plasma or microparticles [6, 7]. Composite serum markers, such as the enhanced liver fibrosis test, have been correlated with skin and lung fibrosis in cross-sectional studies [8]. Discovery approaches have identified new serum markers and components of innate immunity using more advanced methods, such as high-resolution protein analysis and mass cytometry [6, 9, 10].

In addition to blood, urine offers a highly relevant and accessible substrate to explore candidate biomarkers of SSc-related CKD (SSc–CKD). Urine potentially has significant advantages over serum or plasma as a fluid for renal biomarker investigation. It is produced in direct contact with the epithelial surface of the organ of interest, meaning that relevant proteins expressed in renal injury might be shed directly into the urine. For this reason, urine has been described as a ‘fluid biopsy’ of the kidney and renal tract [11]. Furthermore, urine can be obtained non-invasively from subjects, typically in larger volumes than are available for serum or plasma. In the present study, we have, for the first time, used multiplex technology to measure simultaneously proteins that are plausible markers of SSc–CKD and have included control samples with non-SSc CKD, healthy controls and cases of SSc with no evidence of CKD.

In the present study, we have analysed candidate urinary proteins in SSc–CKD and compared them with serum levels of these analytes. To determine which urinary proteins might be relevant specifically to SSc–CKD by reflecting local molecular pathology, we have compared levels in urine, after correction for urinary creatinine, for SSc, SSc–CKD and non-SSc–CKD patients. In this way, we expected to differentiate potential markers of SSc pathobiology in the kidney that might be useful in clinical trials and also provide insight into the likely mechanisms of CKD that occur in SSc and might impact on clinical outcomes, such as susceptibility to, or recovery after acute kidney injury occurring in the context of scleroderma renal crisis (SRC).

## Methods

### Selection of candidate serum and urine markers

Candidate markers of renal involvement to measure in urine and serum of patients with SSc and controls were defined from previous literature and current concepts of the molecular pathology of SSc and other related multi-system autoimmune rheumatic diseases. The aim was to have a selection of proteins that would help to identify inflammatory, fibrotic and vasculopathic processes in the renal parenchyma that would add to conventional assessment of renal abnormalities, such as serum creatinine and GFR. The following markers were selected for measurement.

IL6 is a likely pathogenic mediator of inflammation and connective tissue dysfunction in SSc. Its expression in urine has been correlated with renal disease in several contexts, including the autoimmune CTD SLE [12]. 

IL18 has been demonstrated to be a mediator of ischaemic damage to the renal tubule in mice [13], and urine concentrations have been validated as a marker of acute kidney injury in humans [14].

TNF-α is a putative mediator of endothelial damage in SSc [15]. Serum and urinary concentrations have been demonstrated to be raised in other forms of nephropathy [16, 17].

VEGF is overexpressed in tissue biopsies and sera from patients with SSc [18]. It is expressed in urine in disease states, and concentrations are independent of the serum concentration [19].

Monocyte chemoattractant proteins 1 (MCP1 or CCL2) and 3 (MCP3 or CCL7) have been described as pathogenic fibroblast activators in SSc [20], and high serum levels have been associated with organ-specific disease activity [21]. Urine concentrations of MCP1 have shown promise as a marker of renal involvement in SLE and diabetic kidney disease [22–24].

Soluble ICAM-1 has been associated with disease severity in SSc in serum and is considered to be a marker of activated endothelium, epithelial cells and fibroblasts [25]. It is expressed and shed in greater quantities in SSc fibroblasts than control fibroblasts [26].

Soluble VCAM-1 has been associated with fibroblast activation and epithelial-to-mesenchymal transition and is a marker of immune cell activation and endothelial cell activation. It is elevated in SRC in other series and has been shown to be increased markedly in the serum in some cases of SRC [25, 27]. Urinary levels have not been examined previously in SSc. 

### Study design and participants

This study was approved by the Royal Free Research and Development team and by the Newcastle and North Tyneside Research Ethics Committee. All individuals provided informed consent for their participation, according to the guidance set out by the research ethics committee.

The study cohort of 79 adult patients attending the scleroderma clinic at the Royal Free Hospital was recruited prospectively. All had definite SSc classified by 2013 ACR/EULAR classification criteria [28]. Kidney function was evaluated using the modification of diet in renal disease estimated glomerular filtration rate (MDRD eGFR) equation [29]. This reflects standard reporting in our centre over the past decade, and we expect that this formula will not differ significantly from the Chronic Kidney Disease Epidemiology Collaboration (CDK-EPI) formula that is also used in other laboratories [30]. Patients with eGFR of <60 ml/min/1.73 m^2^ or <90 ml/min with persistent urinary blood or protein on dipstick were categorized as having CKD, consistent with CKD stages 2–5 in the 2002 guidelines by the Kidney Disease Outcomes Quality Initiative (KDOQI) [31]. By including SSc samples without CKD and non-SSc CKD groups, we hoped that we would identify urinary proteins that are specifically elevated in SSc–CKD and which might reflect the molecular pathology of SSc in CKD. Of the cohort, 40 patients with SSc were classified as no CKD and 39 patients with SSc and CKD. As controls, 11 patients with CKD attributable to other causes (without nephrotic-range proteinuria) were recruited from the general nephrology clinic at the Royal Free Hospital. A further 12 healthy controls, with no diagnosis of SSc and normal renal function, were also included.

### Clinical data

In addition to demographic data (age, sex and ethnicity), the medical history was recorded. For the SSc groups, this included disease-specific organ complications, skin subgroup (limited or diffuse) and the disease-specific ANA. These data are summarized in Table 1.

**Table 1:** Description of study cohort

Parameter	SSc–no CKD (*n* = 40)	SSc–CKD (*n* = 39)	History of SRC (*n* = 14)	CKD (*n* = 11)	Control (*n* = 12)
Age, years	57 (2.1)	63 (1.7)	–	58 (5.1)	34 (2.4)
eGFR, ml/min/1.73 m^2^	80 (1.5)	45 (2.3)	–	49 (7.7)	87 (1.3)
dcSSc, *n* (%)	10 (25)	20 (51)	–	–	–
lcSSc, *n* (%)	30 (75)	19 (49)	–	–	–
ACA, *n* (%)	13 (33)	12 (30)	0	–	–
ATA (Scl-70), *n* (%)	8 (20)	2 (5)	1	–	–
ARA (RNApol), *n* (%)	6 (15)	12 (30)	7	–	–
AFA (U3RNP), *n* (%)	0	3 (8)	2	–	–
Other ANA, *n* (%)	13 (33)	11 (27)	4	–	–

Data are shown as the mean (s.e.m.) or number (percentage within study subgroup).

eGFR: estimated glomerular filtration rate; RNApol: anti-RNA polymerase antibody; SRC: scleroderma renal crisis; U3RNP: anti-fibrillarin autoantibody; ATA: anti-topoisomerase.

### Sample collection and management

From each individual recruited to the study, concurrent urine and blood samples were collected. Clotted blood and fresh mid-stream urine were centrifuged at 600 *g* at 4°C, for 10 min, within 1 h of collection. After centrifuge and serum separation, the serum and urine were divided into aliquots and frozen at −80°C. 

Additional blood and urine samples were sent to the Royal Free Hospital laboratories for routine clinical biochemistry analysis, including serum creatinine, eGFR and urinary albumin:creatinine ratio.

### Multiplex analysis of serum and urine

Multiplex immunoassay for all eight analytes was performed according to the manufacturer’s protocol (Luminex Corporation, Austin, TX, USA). Standards supplied with the assay kits, urine and serum samples were analysed in duplicate wells. Urine biomarker concentrations (in picograms per millilitre) were expressed as a ratio to the urine creatinine concentration (in micromoles per litre) to compensate for diurnal variations in the water concentration of spot urine samples.

### Statistical analysis

Four patient groups (SSc–CKD, SSc–no CKD, non-SSc CKD and control) were compared for each candidate biomarker. The difference between these groups was assessed using the Kruskal–Wallis test. The SSc–CKD group was also compared individually with each of the other three groups using the Mann–Whitney *U*-test. Correlation between eGFR and biomarker concentrations was assessed using Pearson’s coefficient. All statistical tests were performed using GraphPad Prism v.8.2.1 for Windows (GraphPad Software, La Jolla, CA, USA).

## Results

**Fig. 1:**
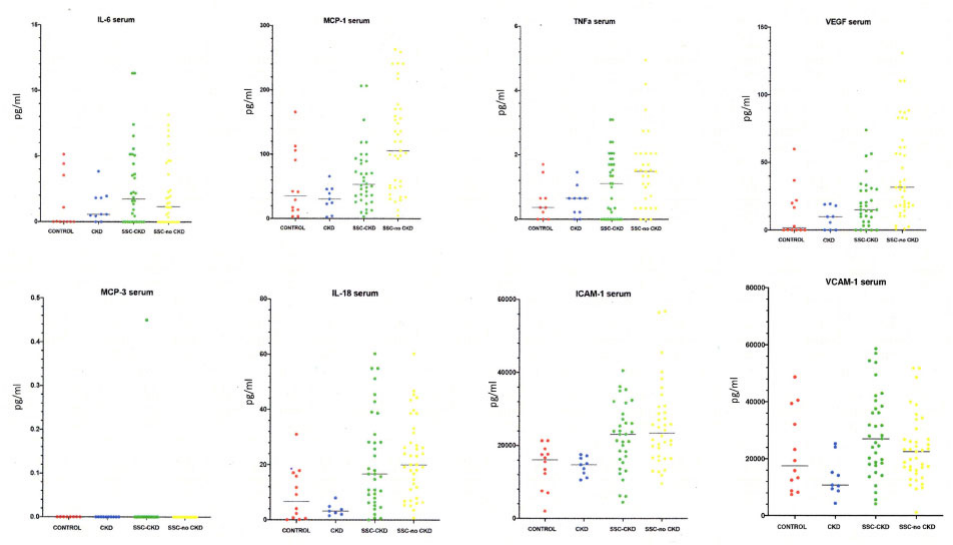
Candidate markers in SSc-associated chronic kidney disease and controls: serum analysis The panels show the distribution for each analyte in serum for the controls and for patients with SSc with chronic kidney disease (CKD) and those without CKD. The panels also show values for the non-SSc cohort with CKD. Significant differences by ANOVA and pairwise comparison are detailed in the main text and in Table 2.

**Fig. 2:**
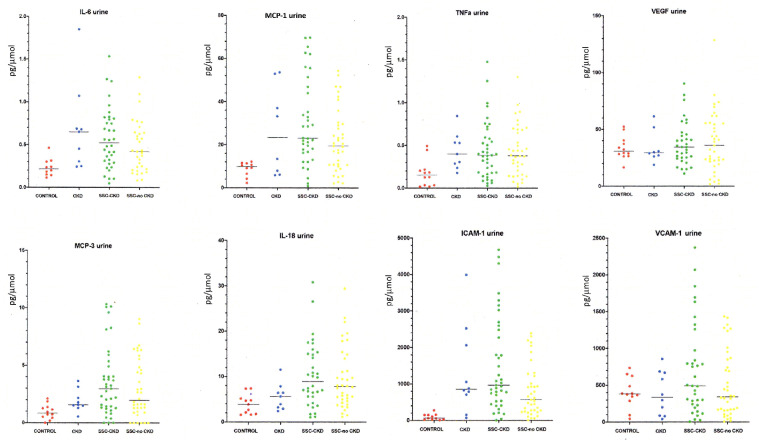
Urinary analyte:creatinine ratio for candidate markers in SSc-associated chronic kidney disease and controls The panels show the distribution for each analyte (analyte:creatinine ratio) for the controls, and for patients with SSc with chronic kidney disease (CKD) and those without CKD. The panels also show values for the non-SSc cohort with CKD. Significant differences by ANOVA and pairwise comparison are detailed in the main text and in Table 3.

Overall results for the eight proteins analysed in this study are detailed in Table 2 for serum and Table 3 for urine. Individual plots for each serum protein are shown in Fig. 1 and urine levels, after correction for urinary creatinine concentration, in Fig. 2.

**Table 2:** Summary of analytes with ANOVA and pairwise comparison for serum

Analyte, pg/ml	HC	CKD	SSc–CKD	SSc–no CKD	ANOVA	SSc *vs* SSc–CKD	SSc–CKD *vs* CKD	SSc–CKD *vs* HC
					*P*-value	*P*-value	*P*-value	*P*-value
IL6								
Median	0	0.57	1.7	1.2	0.3748	0.5614	0.3041	0.133
Minimum	0	0	0	0				
Maximum	5.1	3.8	11	8.2				
Mean	1.3	1.1	2.7	2.1				
s.d.	2	1.2	3.1	2.5				
MCP1								
Median	35	30	53	106	0.0001	0.0015	0.0236	0.2854
Minimum	3.4	2.5	3.4	5.1				
Maximum	166	66	207	263				
Mean	53	31	66	118				
s.d.	53	21	49	76				
TNF-α								
Median	0.36	0.65	1.1	1.5	0.0196	0.1393	0.2136	0.2231
Minimum	0	0	0	0				
Maximum	1.7	1.5	3.1	5				
Mean	0.54	0.56	1.1	1.5				
s.d.	0.6	0.47	0.98	1.2				
VEGF								
Median	1.6	9.9	15	32	<0.0001	0.001	0.0963	0.1453
Minimum	0	0	0	1				
Maximum	60	19	74	131				
Mean	13	9.1	20	43				
s.d.	19	8.3	18	34				
MCP3								
Median	0	0	0	0	NA			
Minimum	0	0	0	0				
Maximum	0	0	0.45	0				
Mean	0	0	0.019	0				
s.d.	0	0	0.092	0				
IL18								
Median	6.6	3.2	17	20	0.0002	0.3991	0.0016	0.0299
Minimum	0	1.5	0	0.72				
Maximum	31	8	60	60				
Mean	9.2	3.7	21	23				
s.d.	9.8	2.2	18	14				
ICAM-1								
Median	16 083	14 762	23 082	23 412	0.0004	0.413	0.0067	0.0079
Minimum	2030	10 564	4453	9580				
Maximum	21 329	17 534	40 541	56872				
Mean	14 287	14 321	22 118	25 449				
s.d.	6055	2554	9090	11 316				
VCAM-1								
Median	17 561	10 749	27 047	22 538	0.0113	0.0832	0.0028	0.1945
Minimum	7436	4373	4167	1196				
Maximum	48 673	25 272	58 652	51 776				
Mean	22 427	13 609	28 734	23 291				
s.d.	14 297	7021	14 814	11 645				

CKD: chronic kidney disease; HC: healthy control; MCP1: monocyte chemoattractant protein 1; MCP2: monocyte chemoattractant protein 2; VCAM-1: vascular cell adhesion molecule 1; ICAM-1: intercellular adhesion molecule 1.

**Table 3:** Summary of analytes with ANOVA and pairwise comparison for urine

Analyte, pg/pmol	HC	CKD	SSc– CKD	SSc–no CKD	ANOVA	SSc *vs* SSc–CKD *P*-value	SSc–CKD *vs* CKD *P*-value	SSc–CKD *vs* HC *P*-value
IL6								
Median	0.22	0.65	0.52	0.42	0.008	0.2243	0.7273	0.0018
Minimum	0.11	0.24	0.048	0.082				
Maximum	0.46	1.9	1.5	1.3				
Mean	0.23	0.69	0.57	0.47				
s.d.	0.1	0.51	0.36	0.3				
MCP1								
Median	9.8	23	23	19	0.0032	0.0868	0.5803	<0.0001
Minimum	2.2	5.9	0.81	2.1				
Maximum	12	54	70	54				
Mean	8.7	26	30	22				
s.d.	3.4	21	20	15				
TNF-α								
Median	0.15	0.4	0.38	0.38	0.0164	>0.9999	0.7162	0.004
Minimum	0.016	0.18	0.028	0.018				
Maximum	0.49	0.84	1.5	1.3				
Mean	0.17	0.44	0.44	0.43				
s.d.	0.16	0.21	0.34	0.3				
VEGF								
Median	31	29	34	36	0.9828	0.9081	0.7654	0.739
Minimum	17	19	11	1.8				
Maximum	52	61	90	129				
Mean	33	34	38	39				
s.d.	10	14	19	27				
MCP3								
Median	0.84	1.6	3	2	0.0101	0.4744	0.2185	0.0008
Minimum	0	0.55	0	0				
Maximum	2.1	3.6	10	9				
Mean	0.94	1.9	3.5	3				
s.d.	0.64	0.95	3	2.6				
IL18								
Median	3.9	5.7	8.9	7.9	0.0053	0.6337	0.0493	0.0026
Minimum	1.6	2.4	1.1	1.2				
Maximum	7.4	12	31	29				
Mean	2.2	2.9	7.1	6.8				
s.d.	0.65	0.97	1.2	1.1				
ICAM-1								
Median	60	855	968	570	<0.0001	0.0134	0.7049	<0.0001
Minimum	8.8	73	9	13				
Maximum	275	3991	4678	2391				
Mean	94	1307	1499	807				
s.d.	82	1211	1302	704				
VCAM-1								
Median	382	336	491	342	0.4377	0.2239	0.198	0.2928
Minimum	44	40	5.2	8.1				
Maximum	735	857	2371	1435				
Mean	400	387	704	509				
s.d.	207	294	632	450				

CKD: chronic kidney disease; HC: healthy control; MCP1: monocyte chemoattractant protein 1; MCP2: monocyte chemoattractant protein 2; VCAM-1: vascular cell adhesion molecule 1; ICAM-1: intercellular adhesion molecule 1.

Seven of the candidate proteins were detectable in the serum of patients with SSc–CKD and control groups. MCP3 was detectable in the serum of only one subject in the SSc–CKD group and none of the subjects in the three control groups and is therefore of limited value for further analysis. All eight candidate proteins were detectable in urine by our method in subjects in all four groups.

Two of the candidate urinary proteins (MCP1 and ICAM-1) showed a higher degree of discrimination between groups, and further analysis was performed to assess whether differences in GFR among our patients would account for the difference in urine concentrations.

### Relationship of candidate urinary markers MCP1 and ICAM-1 with renal function

**Fig. 3:**
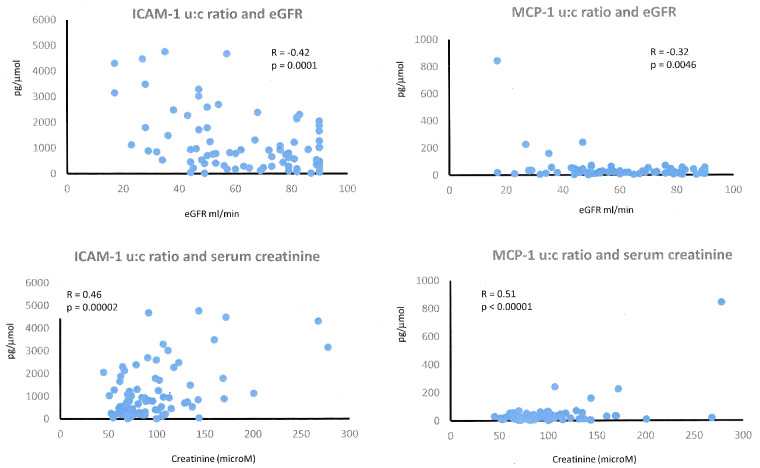
Relationship of candidate urinary markers of SSc-associated chronic kidney disease with renal function The panels show the relationship between renal function estimated by serum creatinine or eGFR compared with urinary MCP1:creatinine and urinary ICAM-1:creatinine ratio. The correlation coefficient (*R*) and *P*-value are annotated for each analysis. eGFR: estimated glomerular filtration rate; MCP1: monocyte chemoattractant protein 1; ICAM-1: intercellular adhesion molecule 1.

The detectable concentration of low molecular weight proteins in the urine is inextricably related to the blood concentration of the same proteins and the volume of blood filtered by the kidneys (i.e. the GFR). To investigate the relationship between kidney function and concentrations of MCP1 and ICAM-1 in urine, urinary concentrations were plotted against MDRD eGFR and serum creatinine (from which the eGFR is derived). These data are included in Fig. 3.

For the ICAM-1:creatinine ratio, there was a significant negative correlation with eGFR (*R* = −0.42, *P* = 0.0001) and positive correlation with serum creatinine (*R* = 0.46, *P* = 0.00002). The correlation for urinary MCP1:creatinine with eGFR followed the same trend but was weaker (*R* = −0.32, *P* = 0.0046). Examination of the dot plots for each marker suggested that the relationship between eGFR and ICAM-1:creatinine was more consistent and robust than that for urinary MCP1. This suggested for MCP1 that raised urinary levels might reflect local pathogenic processes rather than simply lower GFR, consistent with widespread expression of MCP1 in SSc renal biopsy specimens [30].

## Discussion

This project is part of an overarching attempt to look for local sites of disease-associated protein expression in a systemic condition. In this way, we delineated potential markers or mediators of molecular pathology in SSc-associated renal disease. By comparing the findings for SSc-related CKD with non-SSc-related CKD we were able to assess molecular markers that reflected SSc, and by focusing on SSc cases with CKD we reasoned that this would reflect the overall disease process of SSc that was relevant to a target organ, the kidney, in the context of a multi-system disease. Thus, we harnessed the strengths of this approach by including non-CKD SSc controls, in which there was unlikely to be relevant renal pathology, and non-SSc CKD, where the chronic renal impairment was likely to have arisen by non-SSc mechanisms. This study design favoured identification of specific markers of SSc–CKD and offered potential insight into SSc pathobiology more generally by providing confidence that the urinary proteins were associated with local functionally relevant pathology of SSc.

In this study, we show that urinary levels of proteins implicated in SSc pathogenesis have potential as biomarkers for detection and surveillance of renal involvement in this multi-system disease. Based on previous studies of serum markers in SSc and emerging data on urinary analytes in other renal diseases, we selected eight candidates to assess in our well-characterized cohort of SSc patients and relevant controls.

Although many of the proteins were elevated in SSc serum compared with controls, this appears to have reflected disease occurring in multiple organs, because there was no clear difference between serum levels in SSc–CKD and SSc–no CKD. In fact, for half of the proteins the average level was higher in SSc–no CKD, suggesting that disease outside the kidneys had most influence on serum levels. Although not statistically significant, it is notable that VCAM-1 levels on average were higher in SSc–CKD, because the levels have been shown previously to be increased in SRC [26, 27].

Our findings confirm previous studies of cytokines and adhesion molecules in SSc patients. These have demonstrated a correlation of the MCP1 level with skin sclerosis and with the change in lung function in clinical trials [32]. However, most of these studies have focused on serum levels, and this is a challenge for a multicompartment disease, such as SSc, where elevated levels can reflect disease in the skin, lung, kidney or other vascular structures. This might explain why it has been difficult to identify strong correlations with lung fibrosis or pulmonary arterial hypertension in general cohorts [21]. In more selected cases, such as those recruited into the scleroderma lung study, there was a correlation between the change in MCP1 and the treatment response [32]. Likewise, cross-sectional studies have shown that in idiopathic pulmonary fibrosis, where organ systems other than the lungs are more likely to be normal, there is a strong predictive value of serum MCP1 for future disease progression [33].

Urinary analytes appear to reflect renal pathology better, evidenced by average levels being greater for SSc–CKD than for SSc–no CKD in six of the eight analytes examined. Although several of the proteins that were increased in SSc–CKD are of interest in SSc pathogenesis, the overall goal of the present study was to identify the most promising markers in the urine that could reflect CKD in SSc and be used as future biomarkers in observational cohort studies or interventional trials. As outlined in the Results section, we have selected MCP1:creatinine and ICAM-1:creatinine as the most promising candidate markers to take forward, because they show the most significant difference across all groups, the highest discrimination from healthy controls and the most potential to differentiate SSc–CKD from other causes of CKD.

There have been reports of correlation of ICAM-1 with skin or lung involvement in SSc although, as confirmed by the serum results in the present study, the relationship to renal involvement compared with other adhesion molecules is less clear [27]. Nevertheless, ICAM-1 has been shown to change over time in some interventional studies, supporting its possible value as a molecular surrogate of the disease process [34]. Although we report the first study of urinary ICAM-1 in SSc, there have been several studies of urinary ICAM-1 in SLE that show elevated levels compared with controls [34]. Although there is an association with the presence of renal involvement in SLE, a recent meta-analysis concluded that the current evidence does not support urinary ICAM-1 as an effective marker of lupus nephritis activity [35].

This is also the first study to investigate concentrations of MCP1 in the urine of patients with SSc. The SSc–CKD group had lower serum and higher urinary concentrations of MCP1 than the SSc–no CKD group. The negative correlation with GFR and histology studies support the proposition that this relates to local chemokine expression in the kidney [36].

MCP1 is a C-C group chemokine produced by many cell types, including endothelial and epithelial cells and fibroblasts [37], but monocytes/macrophages are the major source [38]. MCP1 has been shown in previous work to promote the differentiation of fibroblasts into myofibroblasts in SSc via its receptor, CCR2 [39]. It is also a chemoattractant for monocytes, T lymphocytes and NK cells [38]. MCP1 has previously been demonstrated to be upregulated in affected areas of SSc skin [39], in bronchoalveolar lavage fluid [39] and in the sera of some SSc patient subgroups [21]. Thus, MCP1 has a plausible role in the pathogenesis or maintenance of organ complications of SSc.

However, attempts to establish the serum MCP1 concentration as a dynamic biomarker of disease have not been successful. One plausible explanation for this is that serum concentrations do not reflect local tissue expression of pathogenic chemokines except where there is a high burden of skin disease [39]. For this reason, the identification of urine as a biomarker fluid that allows assessment of local expression of MCP1 or other pathogenic mediators is a potentially significant development in the management of SSc.

Consistent with our findings in CKD, there is evidence from juvenile onset SLE that active renal involvement is associated with increased urinary levels of MCP1 and that levels in SLE are higher than those in matched healthy controls [22, 23, 40]. In adult SLE, a combined assessment of urinary MCP1 and urinary TNF related weak inducer of apoptosis (TWEAK) has been proposed as an early marker of nephritis, raising the possibility of combining urinary markers to improve performance in detecting a preclinical disease state [41]. This is consistent with recent results that increased urinary MCP1 might precede and predict drug-induced renal toxicity [42].

A particular strength of our study is the inclusion and careful stratification of three control groups: SSc–no CKD, CKD–no SSc and healthy controls. The CKD–no SSC controls were selected to have non-proteinuric underlying diseases in order that these could serve as controls for reduced GFR without the confounding of non-selective glomerular protein leak.

Limitations include the small number of samples and the cross-sectional design. This reflects the relative rarity of SSc and the need for well-characterized patients in discovery studies looking for new potential molecular markers of disease. Nevertheless, we consider that the number of cases was sufficient to include most of the major patterns and subsets of SSc as evidenced by the serological and clinical features of our study cohort.

Another limitation is the categorization of cases of SSc as SSc–CKD, because this inevitably includes a wide range of severity of renal involvement and multiple potential mechanisms of renal disease. This might explain the diversity of results for some analytes, including MCP1. It is possible that a threshold level might be important, and future studies could compare cases with high- and low-level urinary MCP1 to explore clinical associations with biomarker expression.

Future work is needed to validate our cross-sectional study and could explore the use of urinary MCP1 and ICAM-1 as longitudinal markers. Such studies could be in observational cohorts and could also examine these new molecular markers in prospective interventional clinical trials. These analytes might act as pharmacodynamic markers or provide evidence of effect on relevant pathobiology in SSc and especially SSc–CKD.

A similar approach has been fruitful in SSc for serum IL6 and lung function decline, in idiopathic pulmonary fibrosis with serum MCP1 predicting respiratory outcome, and in SSc-associated pulmonary arterial hypertension (PAH), where MCP1 showed relevant changes after treatment [21]. Likewise, serum ICAM-1 has shown response to experimental treatment in liver disease [43].

In summary, we have taken a new approach to identify potential urinary protein markers of SSc–CKD. Two promising candidates have been identified, and these should be validated in future cross-sectional and longitudinal studies.

*Funding*: This work was funded by the Medical Research Council (award reference MR/K015230/1) and performed as part of an academic collaboration with AstraZeneca. Additional funding was provided by Scleroderma and Raynaud’s UK.

*Disclosure statement*: R.U. is currently working in Early Clinical Development, Early CVRM (Cardiovascular, Renal and Metabolism), R&D BioPharmaceuticals, AstraZeneca, Cambridge, UK and Gothenburg, Sweden. The other authors have declared no conflicts of interest.

## Data availablility statement

The authors agree to make all data, available materials and associated protocols available to readers if requested for academic purposes.
